# Cancer du cavum d’emblee metastatique: étude clinique et pronostique (à propos de 51 cas)

**DOI:** 10.11604/pamj.2018.29.155.11257

**Published:** 2018-03-15

**Authors:** Noureddine Bouaouina, Sarra Ouni, Samia Belajouza Kanoun, Abir Ben Neffeti, Wassim Kermani, Mohamed Abdelkefi

**Affiliations:** 1 Service de Carcinologie Radiothérapie, CHU Farhat Hached, Sousse, Tunisie; 2 Service d’Oto-Rhino-Laryngoglogie, CHU Farhat Hached, Sousse, Tunisie

**Keywords:** Cancer du nasopharynx, métastase, radiothérapie, chimiothérapie, Nasopharynx cancer, metastasis, radiation therapy, chemotherapy

## Abstract

L’objectif de cette étude rétrospective était de détailler les différents aspects épidémiologiques, cliniques, thérapeutiques et pronostiques du cancer du cavum d'emblée métastatique dans le centre Tunisien. Il s’agit d’une étude portant sur 51 patients atteints d’un cancer du nasopharynx (CNP) histologiquement prouvé et métastatique d’emblée, soit au moment du diagnostic , colligés dans les services ORL du CHU Farhat Hached de Sousse et les services de radiothérapie du centre tunisien, à savoir du CHU Farhat Hached de Sousse, et du centre médical Ibn Khaldoun de Hammam Sousse, entre Janvier 1995 et Décembre 2010. Cinquante et un patients atteints de cancer du nasopharnx, d’emblée métastatiques ont été recensés. L’âge moyen était de 49 ans. Le Sex-Ratio était de 6,2. Les métastases osseuses étaient les plus fréquentes (94,1%), suivies par les métastases hépatiques (34,6%). Sur le plan thérapeutique, la majorité de nos patients a reçu une chimiothérapie (41 patients), dont 31 en association avec une radiothérapie. Le protocole adriamycine et cisplatine a été le plus fréquemment utilisé (92,6%). Vingt patients ont eu une radiothérapie cervicofaciale à intention curative (doses ≤ 70 Gy), quatorze autres patients ont reçu au niveau du cavum une radiothérapie à visée symptomatique ( doses de 30Gy en 10 séances) associée à une irradiation des sites métastatiques dans 9 cas. Les survies globales à 2 ans et à 5 ans étaient respectivement de 29% et de 10%. Au stade de métastases inaugurales, le cancer du cavum est une maladie grave, rapidement mortelle malgré les avancées thérapeutiques. Au vue des données de notre étude et de celles de la littérature, la chimiothérapie à base de cisplatine et la radiothérapie cervico-faciale à doses curatives et celle des métastases a permis d’obtenir de longues survies et même des réponses complètes. Les futurs essais pourraient tester de nouvelles molécules en vue d'une intensification thérapeutique.

## Abstract (En)

This retrospective study aimed to highlight the different epidemiological, clinical, therapeutic and prognostic features of nasopharinx cancer with inaugural metastases in patients hospitalized in a Tunisian Hospital. We here report 51 cases of histologically diagnosed nasopharynx cancer (NPC) with inaugural metastases. Data were collected in the ENT departments and in the radiation therapy departments at the University Hospital Farhat Hached, Sousse, Tunisia as well as at the Centre Medical Ibn Khaldoun, Hammam Sousse, between January 1995 and December 2010. We collected data on 51 patients with metastatic nasopharynx cancer at diagnosis. The average age was 49 years. Sex ratio was 6,2. Bone metastases were the most common finding (94.1%), followed by liver metastases (34.6%). In the majority of our patients therapeutic approach was based on chemotherapy (41 patients); 31 of these patients underwent chemotherapy in combination with radiation therapy. Adriamycin+cisplatin protocol was the most frequently used (92.6%). Twenty patients underwent cervicofacial radiation therapy with curative purposes (doses ≤ 70 Gy), fourteen patients underwent radiation treatment to the nasopharynx with symptomatic purposes (doses of 30Gy in 10 sessions) associated with radiation treatment to the metastatic sites in 9 cases. Overall survival at 2 years and 5 years was 29% and 10%, respectively. Nasopharinx cancer with inaugural metastases is a serious disease, rapidly fatal despite therapeutic advances. In view of our data and of those from the literature cisplatin-based chemotherapy and cervicofacial radiation therapy with curative doses and that of metastases resulted in long-term survival and even in complete responses. Future trials should focus on new molecules for therapeutic intensification.

## Introduction

Les carcinomes nasopharyngés représentent une entité spécifique différente du reste des cancers de la tête et du cou. En Tunisie, le cancer du cavum représente le deuxième cancer de la tête et du cou après le cancer du larynx. Le diagnostic du cancer du cavum a énormément bénéficié durant ces dernières années des progrès de l’endoscopie, de l’imagerie tomodensitométrique, par résonnance magnétique et des explorations isotopiques précisant par ailleurs son extension [1]. Le diagnostic reste tout de même tardif avec des formes évoluées, voire même métastatiques au moment du diagnostic dans moins de 5% des cas [2]. Ce cancer présente au cours de son évolution un taux élevé de métastases ganglionnaires et viscérales, qui expliquent une partie des échecs thérapeutiques, malgré une chimiosensiblité et une radiosensibilité marquées, qui permettent de contrôler avec une fréquence relativement élevée la tumeur primitive. La radiothérapie constitue le pilier du traitement de ce cancer au stade non métastatique, cependant en phase métastatique, celle-ci a été peu étudiée. Le rôle de la chimiothérapie dans cette situation est bien établi actuellement, attesté par un taux élevé de réponses objectives avec des rémissions durables et quelques longues survies [3]. L’objectif de ce travail était de détailler les différents aspects épidémiologiques, cliniques, thérapeutiques et pronostiques du cancer du cavum d'emblée métastatique au centre tunisien.

## Méthodes

Il s’agit d’une étude rétrospective ayant concerné 51 patients atteints d’un cancer du nasopharynx histologiquement prouvé et métastatique d’emblée, au moment du diagnostic ini- tial, colligés dans les services ORL du CHU Farhat Hached de Sousse et les services de radiothérapie du centre tunisien, à savoir du même CHU , et du centre médical Ibn Khaldoun de Hammam Sousse, entre Janvier 1995 et Décembre 2010. L'analyse statistique a été réalisé à l'aide du logiciel SPSS version 18.0.

## Résultats

### Aspects épidémiologiques et cliniques

L’âge moyen de nos patients était de 49 ans avec des extrêmes entre 10 et 78 ans. Une prédominance masculine était noté avec un sex-ratio de 6,2 (Tableau 1). Sept patients avaient des antécédents familiaux de cancers. Dans 4 cas, il s’agissait d’un cancer du cavum et dans les 3 cas restants, d’un cancer du sein, du colon et du larynx. Près de 24% des malades présentaient des signes cliniques liés à la présence de métastases au moment de la première consultation. Il s’agissait de douleurs osseuses dans 91,66% des cas et abdominales dans 8,33%. Par ailleurs, nous avons noté que 33 patients (soit 64,7%) avaient des métastases uniques: osseuses chez 31 patients et hépatiques dans 2 cas. Dix-huit patients (soit 35,2%) avaient des métastases multiples, avec une association plus marquée pour l’atteinte osseuse et hépatique.

**Tableau 1:** Critères épidémiologiques et cliniques

	Pourcentage (%)
**Sexe**	
masculin	86
Féminin	14
**Age (ans)**	
<20	3,9
20-50	47,1
>50	49
**Symptômes**	
Adénopathies cervicales	92,15
Signes otologiques	41,2
Signes rhinologiques	56,8
Signes neurologiques	43,1
Signes liés aux métastases	23,5
**Etat général**	
OMS 1-2	78
OMS3-4	22
**T classification (2002)**	
T1/T2/T3/T4	12,4/33/19,6/33
**N classification (2002)**	
N0/N1/N2/N3	8/12/38,2/41,8
**Site métastatique**	
Os	94,1
foie	34,6
poumon	7,8
rate	3,9
Moelle osseuse	1.9
Métastase unique	64,7
Métastase multiple	35,2

### Sur le plan thérapeutique

**Figure 1:**
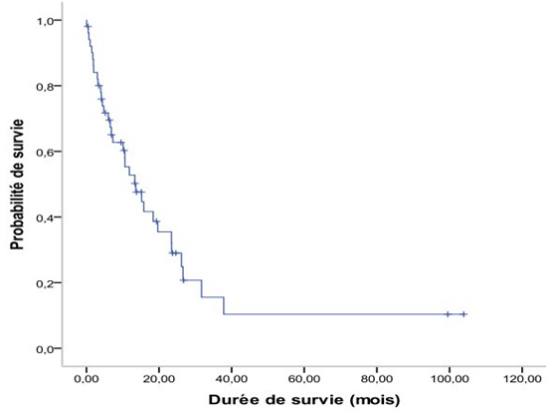
La survie globale

La majorité de nos patients ont reçu une chimiothérapie (41 patients), dont 31 en association avec une radiothérapie. Le protocole adriamycine et cisplatine a été le plus fréquemment utilisé (92,6 %). Vingt patients ont eu une radiothérapie cervicofaciale à intention curative à des doses =70 Gy en étalement fraction classique. Quatorze autres patients ont reçu au niveau du cavum une RT à intention purement palliative, à visée symptomatique, à la dose de 30Gy en 10 séances, associée à une irradiation des sites métastatiques dans 9 cas. Seulement 3,9% des patients ont eu une radiothérapie cervicofaciale avec une chimiothérapie concomitante, 10 malades ont reçu une chimiothérapie seule et cinq autres ont eu uniquement des soins oncologiques de support. Au niveau du site tumoral, on a noté une réponse complète dans 3 cas (7,3%) et une réponse partielle dans 23 cas (56,1%). Le reste des patients était en échec thérapeutique. Au niveau des sites métastatiques, seulement 36 patients ont été évalués, on a noté une réponse complète dans 10 cas, une réponse partielle dans 13 cas et les 13 cas restants étaient en échec thérapeutique. Les survies globales à 2 ans et à 5 ans étaient respectivement de 29 % et de 10% (Figure 1).

## Discussion

L’incidence du carcinome nasopharyngé augmente pendant l’adolescence avec un pic entre 50-59 ans dans les zones à haut risque (Sud-est de l’Asie) alors que dans les pays à risque faible (Etats-Unis), l’incidence atteint son apogée tardivement (=65 ans) [4]. En Afrique, 17% des cancers du cavum surviennent à un âge jeune (<20 ans) , alors que le pourcentage est moins de 5% dans le sud de la Chine [5]. L’incidence en Afrique suggère une distribution bimodale de l’âge avec un premier pic à l’âge de 16 ans et un deuxième pic à l’âge de 40-50 ans [6,7]. Dans les séries faites sur la forme d’emblée métastatique, l’âge moyen des patients était de 46 ans [8,9]. Dans notre série, un pic de fréquence a été noté dans la tranche d’âge de 41-60 ans, soit 55% des patients. Chez nos patients, les localisations métastatiques étaient représentées par ordre de fréquence par l'os, le foie, le poumon et la rate. Ces résultats se rapprochent de ceux de la majorité des séries publiées [10-12]. Fandi et al [13] ont montré que l’atteinte médullaire était fréquente (23%). Elle est liée aux métastases osseuses et est un facteur de pronostic défavorable. Sur le plan thérapeutique, la chimiothérapie constitue le traitement de base dans la prise en charge des cancers nasopharyngés métastatiques. Les données de son efficacité ont largement été rapportées par plusieures études [10,14,15]. Les résultats des polychimiothérapies à base de sels de platine se sont avérés supérieurs à ceux des monochimiothérapies ou des polychimiothérapies sans sels de platine, en termes de réponses objectives et de réponses complètes de longue durée [13,14,16].

Par ailleurs, dans les formes d’emblée métastatiques du carcinome nasopharyngé, l’application de la radiothérapie au niveau du site tumoral primitif, soit le cavum, a été longtemps controversée [17]. Toutefois, grâce aux progrès des techniques d’irradiation, ainsi que l’efficacité des combinaisons de chimiothérapie à base de platine, certaines études ont montré que le contrôle local de la tumeur, tributaire de l’action de la radiothérapie, a amélioré la qualité de vie des patients et a permis d’obtenir de longues survies qui peut aller jusqu'à 91 mois en cas d'association de radio chimiothérapie [18]. En effet, dans l’étude de Yeh et al [17], portant sur 125 cas de CNP d’emblée métastatique, colligés entre M ars 1993 et Septembre 2001, la radiothérapie cervico-faciale ressortait comme un facteur pronostique indépendant, influençant la survie globale des patients . Ainsi, les données fournies par cette étude ont appuyé l’idée que la radiothérapie au niveau du site tumoral offrait un certain bénéfice aussi bien en terme de contrôle locorégional que de survie. De même, dans une autre étude menée par Zeng et al [19] et portant sur 234 cas de CNP métastatique dont 140 traités par chimiothérapie suivie d'une radiothérapie cervico-faciale, les auteurs ont montré que la radiothérapie cervico-faciale a amélioré significativement la survie des patients , avec un taux de survie à 3 ans de 59,6%.

Il en ressort que pour les patients métastatiques d’emblée, une radiothérapie cervicofaciale peut être indiquée après contrôle de la maladie métastatique, permettant ainsi un meilleur contrôle de la maladie et une meilleure qualité de vie [20]. Ailleurs, la radiothérapie des sites métastatiques a comme but de soulager les symptômes tels que la douleur, la compression… [19]. En effet la radiothérapie des métastases osseuses constitue le traitement de choix dans cette situation. Elle a pour but de soulager les douleurs osseuses, de prévenir les fractures osseuses et les tassements vertébraux en consolidant l’os pathologique et de lever les compressions nerveuses [21]. Elle permet ainsi une amélioration symptomatique dans 80% des cas et une sédation des douleurs chez près de la moitié des patients [22]. Une dose de 30 Gy délivrée en dix séances est un schéma qui semble être un bon compromis entre l’efficacité et la tolérance de cette irradiation, et qui a permis d’obtenir des rémissions durables [14,22].

D’ailleurs, en matière de CNP d’emblée métastatique, des survies prolongées supérieures à 2 voire 5 ans, ont été rapportées, particulièrement en cas de métastases osseuses isolées traitées par chimiothérapie et radiothérapie à leur niveau [16]. Dans notre série, la survie globale à 2 ans est de 29%, d’autres auteurs ont rapporté des survies de longue durée, jusqu’à 155 mois. La quasi-totalité des patients dans ces séries a reçu une polychimiothérapie à base de sels de platine et la majorité d’entre eux a été irradiée ou opérée des localisations métastatiques [16].

Il ressort d’après nos résultats et de ceux de Fandi et al. que les facteurs de prédiction d’une longue survie seraient une oligométastase osseuses (moins de trois lésions osseuses) et le traitement locorégional ainsi que celui des métastases [16].

## Conclusion

Au stade de métastases inaugurales, le cancer du cavum est une maladie grave, rapidement mortelle malgré les avancées thérapeutiques. Au vue des données de la littérature et celle de notre étude, la chimiothérapie à base de cisplatine et la radiothérapie cervico-faciale à doses curatives et celle des métastases a permis d’obtenir de longues survies et même des réponses complètes. Les futurs essais pourraient tester de nouvelles molécules en vue d'une intensification thérapeutique.

### Etat des connaissances actuelles sur le sujet

- Le cancer du cavum métastatique au moment de diagnostic est une maladie grave, rapidement mortelle;
- Une polychimothérapie est le traitement standard dans ce cas.

### Contribution de notre étude à la connaissance

- Même au stade métastatique une irradiation cervico-faciale a un impact sur le contrôle locorégionale ainsi que la survie;
- Les métastases osseuses sont les plus fréquentes, et elles sont les plus répondeuses sur le plan thérapeutique.

## Conflits d’intérêts

Les auteurs ne déclarent aucun conflit d'interêts.
